# Early Postweaning Treatment with Dimethyl Fumarate Prevents Prenatal Dexamethasone- and Postnatal High-Fat Diet-Induced Programmed Hypertension in Male Rat Offspring

**DOI:** 10.1155/2018/5343462

**Published:** 2018-02-15

**Authors:** Yu-Ju Lin, I-Chun Lin, Hong-Ren Yu, Jiunn-Ming Sheen, Li-Tung Huang, You-Lin Tain

**Affiliations:** ^1^Department of Obstetrics and Gynecology, Kaohsiung Chang Gung Memorial Hospital and Chang Gung University College of Medicine, Kaohsiung, Taiwan; ^2^Department of Pediatrics, Kaohsiung Chang Gung Memorial Hospital and Chang Gung University College of Medicine, Kaohsiung, Taiwan; ^3^Institute for Translational Research in Biomedicine, Kaohsiung Chang Gung Memorial Hospital and Chang Gung University College of Medicine, Kaohsiung, Taiwan

## Abstract

Prenatal dexamethasone (DEX) exposure, postnatal high-fat (HF) intake, and oxidative stress are closely related to the development of hypertension. Nuclear factor erythroid-derived 2-related factor 2 (Nrf2) regulates oxidative stress. Dimethyl fumarate (DMF) reportedly activates Nrf2 and protects against oxidative stress damage. We examined a 4-month-old male rat offspring from five groups (*n* = 8 for each group): control, DEX (0.1 mg/kg i.p. from a gestational age of 16 to 22 days), HF (D12331 diet from weaning to 4 months of age), and DEX + HF, DEX + HF + DMF (50 mg/kg/day via gastric gavage for 3 weeks after weaning). We found that postnatal HF intake aggravated prenatal DEX-induced hypertension in adult male offspring, which could be prevented by DMF treatment. The beneficial effects of DMF treatment include an increase in renal *Nrf2* gene expression, reduction of oxidative stress, decrease in plasma asymmetric dimethylarginine (ADMA) and renal soluble epoxide hydrolase protein levels, increase in the l-arginine-to-ADMA ratio, and activation of genes related to nutrient sensing and autophagy (e.g., *Pparb*, *Pparg*, *Ppargc1a*, *Ulk1*, and *Atg5*). In conclusion, better understanding of the impact of the Nrf2 signaling pathway in the two-hit model will aid in protecting children exposed to antenatal corticosteroids and a postnatal HF diet from programmed hypertension.

## 1. Introduction

Both pre- and postnatal environmental insults have an influence on developmental programming, leading to the development of hypertension in adulthood through a process known as “developmental origins of health and disease” (DOHaD) [1]. For example, even though glucocorticoids are recommended to women at risk of preterm birth to accelerate fetal lung maturation [2], a growing body of evidence indicates that prenatal glucocorticoid exposure induces a variety of adult diseases, including hypertension [3–5]. The regulation of blood pressure (BP) is a complex process, primarily governed by the kidneys. During nephrogenesis, early-life insults have an influence on renal structural and functional development through a process known as renal programming [6, 7]. A previous study of ours showed that prenatal dexamethasone (DEX) exposure induces hypertension in adult offspring, which is driven by renal programming [8]. Additionally, certain postnatal nutritional insults can exacerbate problems in the programming process. Our recent work further demonstrated that a postnatal high-fat (HF) diet increases offspring susceptibility to prenatal DEX-induced programmed hypertension [9, 10].

Emerging evidence suggests that oxidative stress is involved in renal programming [11, 12]. An imbalance in the nitric oxide- (NO-) reactive oxygen species (ROS) is a particularly important mechanism for programmed hypertension [13]. Nuclear factor erythroid-derived 2-related factor 2 (Nrf2) is a key transcription factor in the regulation of several genes involved in the oxidative stress response. In a spontaneously hypertensive rat, the development of hypertension is related to impaired Nrf2 signaling, which can be attenuated by resveratrol, an Nrf2 activator [14]. We recently found that resveratrol is also involved in mediating the nutrient-sensing signaling pathway for the prevention of maternal and postweaning HF-induced hypertension [15]. Furthermore, we have also previously observed that DEX + HF induces hypertension concurrently with increases in both renal protein levels and the activity of soluble epoxide hydrolase (SEH) in adult offspring, which is prevented by SEH inhibition [10]. Because glucocorticoid can inhibit the Nrf2-dependent antioxidant response and the Nrf2 activator can prevent HF diet-induced obesity and related diseases [15–17], we hypothesize that the activation of Nrf2 could be a therapeutic approach in the prevention of prenatal DEX- and postnatal HF-induced programmed hypertension via the regulation of oxidative stress, SEH, and nutrient sensing.

A variety of Nrf2 pathway-activating agents, including dimethyl fumarate (DMF), have been tested for in several diseases and disorders [18]. DMF has been reported to activate Nrf2 and thus protect the kidney against damage from oxidative stress [19]. Although Nrf2 is involved in BP regulation [20], the antihypertensive effects of DMF are still not well understood. Therefore, the present study tested 2 hypotheses: (1) a postnatal HF diet exacerbates prenatal DEX-induced programmed hypertension through disruption of oxidative stress, SEH, and nutrient-sensing signaling pathways and (2) early postnatal treatment with DMF can prevent programmed hypertension in this two-hit model.

## 2. Material and Methods

### 2.1. Animal Models

This study was approved by the Institutional Animal Care and Use Committee of the Kaohsiung Chang Gung Memorial Hospital. All animal experiments were carried out in a facility accredited by the Association for Assessment and Accreditation of Laboratory Animal Care International (AAALAC) in strict accordance with the recommendations of the Guide for the Care and Use of Laboratory Animals of the National Institutes of Health. Virgin Sprague-Dawley (SD) rats (12–16 weeks old) were obtained from BioLASCO Taiwan Co., Ltd. (Taipei, Taiwan). The rats were exposed to a 12 h light/12 h dark cycle. Male SD rats were housed with individual females until mating was confirmed by the examination of a vaginal plug. To construct a prenatal DEX exposure model, dexamethasone (0.1 mg/kg body weight (BW)) was intraperitoneally administered to pregnant SD rats from a gestational age of 16 to 22 days [4, 8]. After their birth, litters were culled to a total of eight pups to standardize the received quantity of milk and maternal pup care. Because hypertension occurs at a higher rate and at an earlier age in males than females [21], only male offspring were selected from each litter and used in subsequent experiments. Male offspring were assigned to five groups (*n* = 8 for each group): control, DEX, HF, DEX + HF, and DEX + HF + DMF. Male offspring rats were administered either a normal diet with regular rat chow (ND; Fwusow Industry Co. Ltd., Taichung, Taiwan; 52% carbohydrates, 23.5% protein, 4.5% fat, 10% ash, and 8% fiber) or a high-fat hypercaloric diet (HF; D12331, Research Diets Inc., New Brunswick, NJ, USA; 58% fat (hydrogenated coconut oil) plus high sucrose (25% carbohydrate)) from weaning to 4 months of age. In addition to DEX and a postnatal HF diet, rats in the DEX + HF + DMF group were administered an oral dose of DMF 50 mg/kg/day via gastric gavage for 3 weeks after weaning (Sigma, St. Louis, MO, USA). The dose of DMF used here was based on a previous study conducted with rats [17]. Systolic BP was measured in conscious rats by using an indirect tail-cuff method (BP-2000, Visitech Systems Inc., Apex, NC, USA) as previously described [22]. Three stable consecutive measures were taken and averaged. All offspring were killed at 16 weeks of age. Rats were anesthetized using an intraperitoneal injection of ketamine (50 mg/kg) and xylazine (10 mg/kg), then euthanized by an intraperitoneal overdose of pentobarbital. Heparinized blood samples were collected at the end of the study. The kidneys were subsequently collected and stored at −80°C for further future analysis.

### 2.2. High-Performance Liquid Chromatography (HPLC)

The levels of several components of the NO pathway, including l-arginine, l-citrulline, asymmetric dimethylarginine (ADMA, an endogenous inhibitor of nitric oxide synthase), and symmetric dimethylarginine (SDMA, an isomer of ADMA), were measured using high-performance liquid chromatography (HP series 1100; Agilent Technologies Inc., Santa Clara, CA, USA) with the o-phtalaldehyde-3-mercaptoprionic acid derivatization reagent described previously [22]. Standards contained concentrations of 1–100 mm l-arginine, 1–100 mM l-citrulline, 0.5–5 mM ADMA, and 0.5–5 mM SDMA. The recovery rate was approximately 95%.

### 2.3. Quantitative Real-Time Polymerase Chain Reaction (PCR)

RNA was extracted from the kidney cortex according to previously described methods [15]. In addition to *Nrf2*, several genes related to the nutrient-sensing signaling pathway and autophagy were analyzed in this study, including peroxisome proliferator-activated receptor (PPAR) *α* (*Ppara*), *β* (*Pparb*), and *γ* (*Pparg*); PPAR*γ* coactivator 1-*α* (encoded by *Ppargc1a*); serine/threonine kinases; UNC-51-like kinase-1 (*Ulk1*); and autophagy-related gene 5 (*Atg5*). R18S was used as a reference in all analyses. Primer sequences are provided in Table 1. To quantify the relative gene expression, the comparative threshold cycle (*C*_T_) method was employed. For each sample, the average *C*_T_ value was subtracted from the corresponding average *Rn18s* value, calculating the *ΔC*_T_. ΔΔ*C*_T_ was calculated by subtracting the average control Δ*C*_T_ value from the average experimental Δ*C*_T_. The fold increase of the experimental sample relative to the control was calculated using the formula 2^−ΔΔ*C*T^.

### 2.4. Western Blot

Western blot analyses were performed as previously described [15]. We used a rabbit antirat SEH antibody (1 : 1000, overnight incubation; Santa Cruz Biotechnology, Santa Cruz, CA, USA) and Ponceau S staining (PonS) to mitigate variations in the total protein loading in western blot. Bands of interest were visualized using ECL reagents (PerkinElmer, Waltham, MA, USA) and quantified by densitometry (Quantity One Analysis software; Bio-Rad), as integrated optical density (IOD) after a subtraction of the background. The protein abundance was represented as IOD/PonS.

### 2.5. Immunohistochemistry Staining

Paraffin-embedded tissue sectioned at 3 *μ*m thickness was deparaffinized in xylene and rehydrated in a graded ethanol series to phosphate-buffered saline. 8-Hydroxydeoxyguanosine (8-OHdG) is a DNA oxidation product that was measured to assess DNA damage. Following blocking with immunoblock (BIOTnA Biotech., Kaohsiung, Taiwan), the sections were incubated for 2 h at room temperature with an anti-8-hydroxydeoxyguanosine (8-OHdG) antibody (1 : 100, JaICA, Shizuoka, Japan), as previously described. Immunoreactivity was revealed using the polymer-horseradish peroxidase (HRP) labelling kit (BIOTnA Biotech.) and 3,3′-diaminobenzidine (DAB) as the chromogen. The sections were then lightly counterstained with hematoxylin and preserved under cover glass. A quantitative analysis of 8-OHdG-positive cells per microscopic field (×400) in the renal sections was performed as previously described [15]. We used a rabbit antirat SEH antibody (1 : 100, overnight incubation; Santa Cruz Biotechnology) for the detection of SEH. A negative control of identical staining omitting incubation with a primary antibody was used.

### 2.6. Statistical Analysis

Data are given as a mean ± SEM. For most parameters, the statistical analysis was done using one-way ANOVA with Tukey's post hoc test for multiple comparisons. BP was analyzed by two-way repeated-measures ANOVA and Tukey's post hoc test. A *P* value of 0.05 was considered statistically significant. All analyses were performed using the Statistical Package for the Social Sciences software (SPSS, Chicago, IL).

## 3. Results

### 3.1. Morphological Features and Blood Pressure

**Figure 1:**
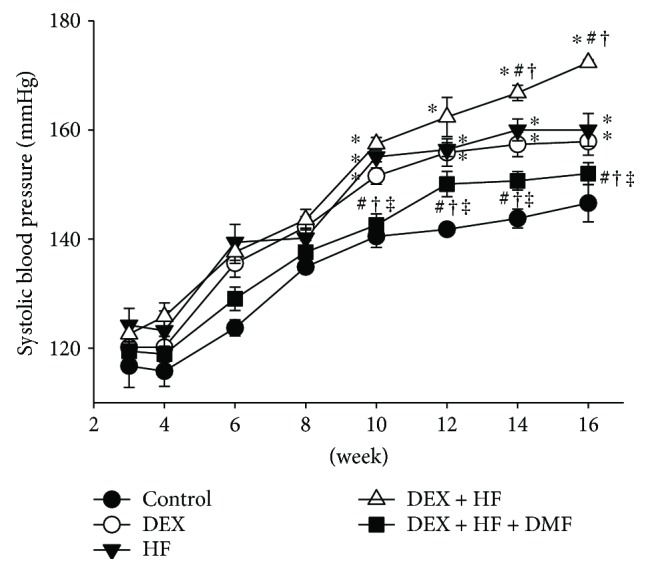
Effects of prenatal dexamethasone (DEX), postnatal high-fat (HF) diet, and dimethyl fumarate (DMF) treatment on systolic blood pressure between 3 and 16 weeks of age. ^∗^*P* < 0.05 versus control; ^#^*P* < 0.05 versus DEX; ^†^*P* < 0.05 versus HF; ^‡^*P* < 0.05 versus DEX + HF.

Litter sizes were not significantly affected by prenatal DEX exposure (pups per litter: control = 12.5 ± 0.6; DEX = 11 ± 1.8). The mortality rate was 0% in each group. The body weight (BW), kidney weight-to-BW ratios, and liver weight-to-BW ratios did not differ among the five groups (Table 2). Prenatal DEX and postnatal HF significantly increased the systolic blood pressure (SBP) in DEX-, HF-, and DEX + HF-exposed animals, which DMF treatment prevented. As shown in Figure 1, the SBP was similar in the five groups at 4 weeks of age. The SBP of the DEX + HF group was higher than that of either the DEX or HF group at 14 and 16 weeks of age. The reduction in the SBP caused by DMF occurred at 10 to 16 weeks of age. Our data indicated a synergistic interaction between the effects of prenatal DEX and postnatal HF on the elevation of the SBP, which was attenuated by DMF treatment.

### 3.2. Oxidative Stress

**Figure 2:**
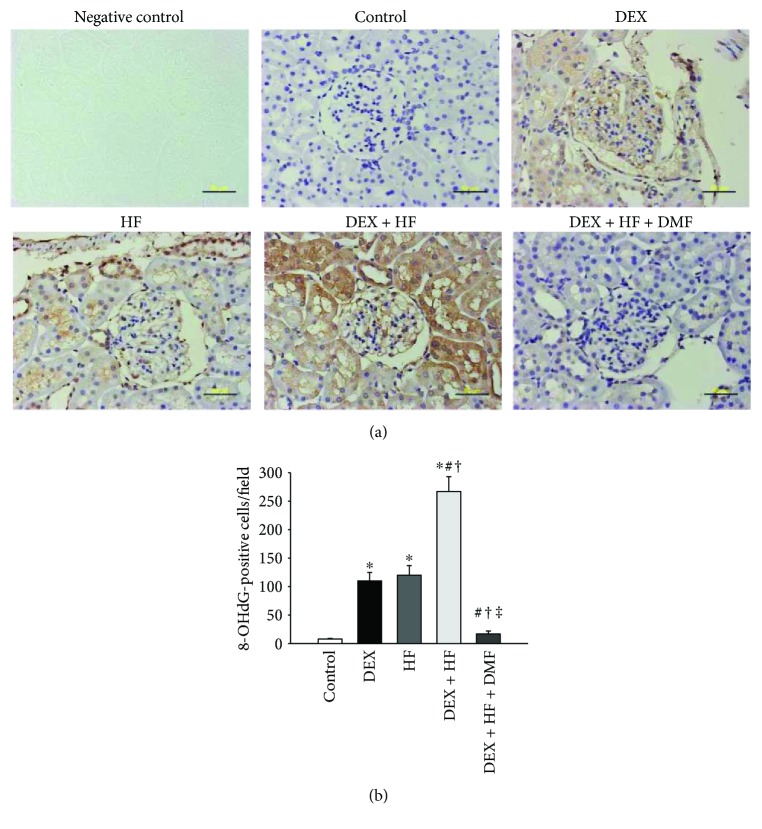
(a) Light micrographs illustrating immunostaining for 8-hydroxydeoxyguanosine (8-OHdG) in the kidney in male offspring at 4 months of age. Bar = 50 *μ*m. (b) Quantitative analysis of 8-OHdG-positive cells per microscopic field (×400). *n* = 5/group. ^∗^*P* < 0.05 versus control; ^#^*P* < 0.05 versus DEX; ^†^*P* < 0.05 versus HF; ^‡^*P* < 0.05 versus DEX + HF.

We evaluated oxidative stress in the kidney by immunohistochemistry for 8-OHdG, a marker of oxidative DNA damage. Immunostaining of both cytoplasmic and nuclear 8-OHdG in the glomeruli and renal tubules indicated intense staining in the DEX + HF group (267 ± 26 positive cells), an intermediate level of staining in the DEX (110 ± 15 positive cells) and HF (120 ± 17 positive cells) groups, and little staining in the control (8 ± 1 positive cells) and DEX + HF + DMF (17 ± 5 positive cells) groups (Figure 2).

Since the link between oxidative stress and the ADMA-NO pathway in programmed hypertension has been previously reported [12, 13], we chose to investigate the ADMA-NO pathway (Table 3). Plasma levels of ADMA and SDMA were higher in the DEX + HF group than that in the control group; their production was then prevented by DMF treatment. The postnatal HF plasma l-arginine-to-ADMA ratio decreased in the offspring of the HF group compared to that of the control group. DMF significantly reversed DEX + HF-induced hypertension and decreased the plasma ADMA and SDMA levels and increased the plasma l-arginine-to-ADMA ratio (Table 3). Once compiled, our findings suggest that DEX + HF-induced renal programming is associated with more ADMA-related oxidative stress, which can be prevented by early DMF treatment.

### 3.3. Nutrient-Sensing Signaling Pathway

**Figure 3:**
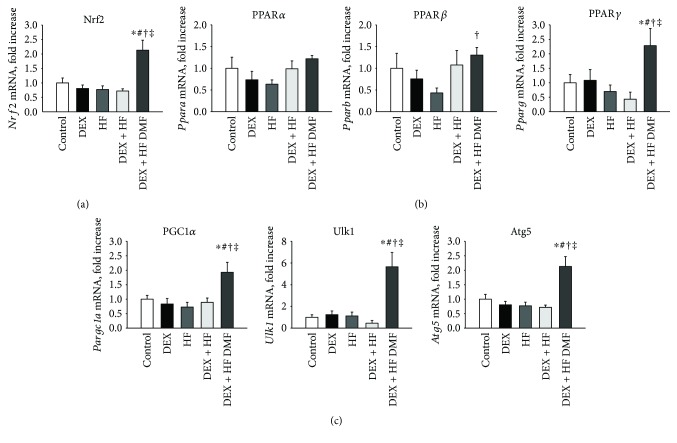
Effects of prenatal dexamethasone (DEX), postnatal high-fat (HF) diet, and dimethyl fumarate treatment (DMF) on mRNA expression of (a) nuclear factor erythroid-derived 2-related factor 2 (*Nrf2*); (b) peroxisome proliferator-activated receptor (PPAR) *α*- (*Ppara*), *β*- (*Pparb*), and *γ*-isoforms (*Pparg*); and (c) autophagy-related genes *Ppargc1a*, *Ulk1*, and *Atg5* in male offspring kidneys at 16 weeks of age. ^∗^*P* < 0.05 versus control; ^#^*P* < 0.05 versus DEX; ^†^*P* < 0.05 versus HF; ^‡^*P* < 0.05 versus DEX + HF.

We analyzed *Nrf2* and other genes involved in the nutrient-sensing signaling pathway in the kidneys of the offspring at 4 months of age (Figure 3). The analysis showed that DMF treatment increased renal mRNA expression of *Nrf2* compared to that of the other groups. Renal mRNA expression of *Pparb* was higher in the DMF group than that in the HF group. DMF treatment significantly increased *Pparg* mRNA expression. Furthermore, we examined whether these genes were involved in autophagy. We observed that there were no significance differences in the mRNA expression levels of *Ppargc1a*, *Ulk1*, and *Atg5* between the control, DEX, HF, and DEX + HF groups. Nevertheless, DMF treatment significantly increased the mRNA expression in the kidneys of the offspring (Figure 3(c)). These results suggest that autophagy is induced by DMF.

### 3.4. SEH Expression

**Figure 4:**
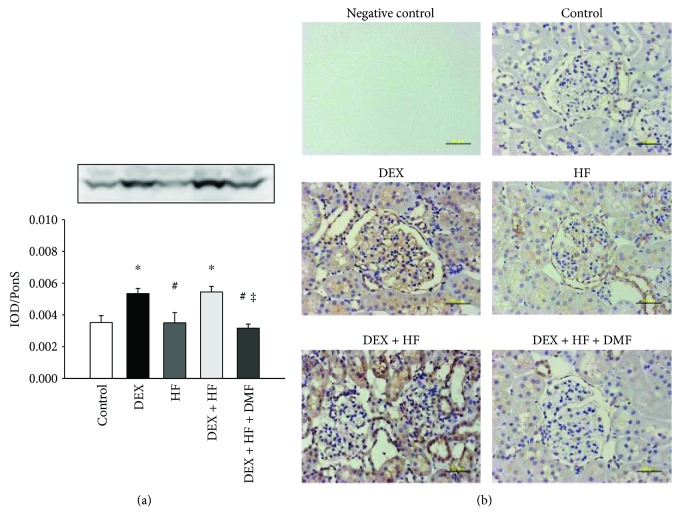
(a) Representative western blot depicting the soluble epoxide hydrolase (SEH) protein (62 kDa). Relative abundance of SEH was quantified by densitometry as integrated optical density (IOD) factored by Ponceau S red (PonS) staining as an internal standard. The protein level is shown as IOD/PonS. (b) Light micrographs illustrating immunostaining for SEH in the kidney in male offspring at 4 months of age. Bar = 50 *μ*m. ^∗^*P* < 0.05 versus control; ^#^*P* < 0.05 versus DEX; ^‡^*P* < 0.05 versus DEX + HF.

We then examined whether SEH levels changed in response to DEX, HF, and DMF in programmed hypertension. As shown in Figure 4, renal SEH protein levels were higher in the DEX and DEX + HF groups compared to that in the control. Renal SEH protein levels were decreased by 42% in the DMF group compared to that in the DEX + HF group (Figure 4(a)). Immunostaining of SEH in the glomeruli and tubules of the kidneys showed significant staining in the DEX and DEX + HF groups and minor staining in the control, HF, and DEX + HF + DMF groups (Figure 4(b)).

## 4. Discussion

The key findings of our study can be summarized as follows: (1) a postnatal HF diet exacerbates prenatal DEX-induced hypertension in adult male offspring; (2) combined DEX exposure and HF diet-induced hypertension relates to elevated plasma ADMA concentration, oxidative stress, and increased SEH protein levels in the kidney; (3) DMF treatment prevented DEX + HF-induced hypertension, which combined with increased renal *Nrf2* gene expression, decreased plasma ADMA levels and renal protein levels of SEH, reduced oxidative stress, and activated nutrient-sensing signaling pathways and autophagy.

The present study is consistent with previous reports showing that prenatal DEX and a postnatal HF diet synergistically induce hypertension in adult offspring [9, 10]. To the best of our knowledge, our study is the first to show that early postweaning DMF treatment can prevent programmed hypertension in this two-hit model. SBP did not decrease with DMF treatment for three weeks after weaning. The antihypertensive effect manifested from ten weeks of age onwards. These findings indicate that any reduction in the SBP after DMF treatment is more likely due to a programming effect than being an acute effect.

Oxidative stress is an important mechanism in a variety of programmed hypertension models [23]. We found an increase in 8-OHdG staining, an oxidative stress damage marker, in the kidneys of offspring exposed to prenatal DEX and a postnatal HF diet; these levels were reduced by DMF treatment. Our data are in agreement with previous studies showing that DMF can activate Nrf2 and protect against oxidative stress damage [19, 24, 25]. Additionally, we found that DMF reversed two-hit-induced increases of ADMA and SDMA concentrations and a decrease in the l-arginine-to-ADMA ratio.

Since ADMA and SDMA inhibit NO production and since the l-arginine-to-ADMA ratio represents NO bioavailability, it can be deduced that DMF reduces ADMA and SDMA, consequently increasing NO bioavailability to prevent the development of hypertension.

In addition to its role in oxidative stress, Nrf2 is believed to regulate BP through alternative mechanisms [20]. The interplay between nutrient-sensing signaling and oxidative stress on the regulation of the PPAR signaling pathway, PGC-1*α*, and autophagy is another mechanism involved in programmed hypertension [23, 26, 27]. As we have previously discussed in other works [26], the PPAR signaling pathway is significantly regulated in a variety of models of programmed hypertension. PPAR*γ* as a nutrient-sensing signal can influence developmental programming of hypertension either directly through PPAR target genes or indirectly through Nrf2 activation [28]. Our current study shows that DMF treatment significantly increased *Pparb* (encoded for PPAR*β*) and *Pparg* (encoded for PPAR*γ*) mRNA expression. Although the activation of both PPAR*β* and PPAR*γ* has been reported for their antihypertensive effects [26, 28–30], further studies are needed to clarify whether the activation of the PPAR signaling pathway is a common protective mechanism of DMF to protect against hypertension in a broad range of programming hypertension models. Additionally, we observed that DMF treatment increased *Ppargc1a* (encoded for PGC-1*α*), *Ulk1*, and *Atg5* mRNA expression. Due to the fact that both PGC-1*α* and the activation of *Ulk1* and *Atg5* have been shown to promote autophagy [31] and because mitochondria are a major source of ROS, our data suggest that selective removal of mitochondria by autophagy might be a protective effect of DMF against two-hit-induced oxidative stress and programmed hypertension.

Our previous study showed that prenatal DEX upregulates SEH in the adult offspring kidney, and SEH inhibition prevents DEX + HF-induced programmed hypertension [8, 10]. In the present study, DMF treatment reduces SEH protein levels in the adult offspring kidney when exposed to DEX + HF. Thus, one might expect the antihypertensive effects of DMF treatment to arise, at least in part, via SEH inhibition. This is consistent with previous reports showing that SEH inhibitors can decrease BP in various animal models of hypertension [32, 33]. Unlike their similar effects on nutrient-sensing signals and oxidative stress markers, prenatal DEX and the postnatal HF diet have contrasting effects on SEH expression. Even though DEX and HF insults induce the same phenotype—that is, hypertension—different insults program a complex set of mechanisms; some pathways have commonality, but others do not. As such, the role of SEH in DEX- or HF-induced hypertension requires further study and clarification.

Our study has few limitations. Firstly, we did not explore different doses or therapeutic windows for resveratrol. Given that programming effects vary during different developmental windows, it would be interesting to explore whether antenatal and postnatal DMF treatment leads to varying levels of protection in programmed hypertension. Secondly, we did not study any of the other organs involved in the regulation of BP. The protective effect of DMF may be attributed to organs other than the kidney. Lastly, we did not conduct a control + DMF group. We did not test DMF in control rats because it already has a favorable safety profile regarding hypotension and hypertension in clinical practice [34]. However, the long-term effects of DMF on normal controls deserve further evaluation.

In conclusion, we found that several important mechanisms are involved in the protective actions of DMF on the kidneys of offspring exposed to prenatal DEX and on a postnatal HF diet, including the reduction of oxidative stress, restoration of an ADMA-related NO/ROS imbalance, SEH inhibition, mediation of the PPAR signaling pathway, and induction of autophagy. Our data suggested a close link between Nrf2, oxidative stress, nutrient-sensing signaling, and SEH underlying the development of hypertension. Additionally, our findings highlight that Nrf2 activation might be a therapeutic approach to prevent hypertension in children exposed to prenatal glucocorticoids and a postnatal HF diet.

## Figures and Tables

**Table 1:** PCR primer sequences.

Gene	Forward	Reverse
*Ppara*	5 agaagttgcaggaggggatt 3	5 ttcttgatgacctgcacgag 3
*Pparrb*	5 gatcagcgtgcatgtgttct 3	5 cagcagtccgtctttgttga 3
*Pparg*	5 ctttatggagcctaagtttgagt 3	5 gttgtcttggatgtcctcg 3
*Ppargc1a*	5 cccattgagggctgtgatct 3	5 tcagtgaaatgccggagtca 3
*Ulk1*	5 gagtacccgcaccagaatgt 3	5 gctgtgtagggtttccgtgt 3
*Atg5*	5 ttggcctactgttcgatcttctt 3	5 ggacagtgcagaaggtcctttt 3
*Rn18s*	5 gccgcggtaattccagctcca 3	5 cccgcccgctcccaagatc 3

**Table 2:** Weights and blood pressures.

	Control	DEX	HF	DEX + HF	DEX + HF + DMF
Mortality	0%	0%	0%	0%	0%
Body weight (BW) (g)	529 ± 9	516 ± 12	523 ± 11	522 ± 11	537 ± 16
Left kidney weight (g)	1.7 ± 0.05	1.77 ± 0.04	1.63 ± 0.06	1.58 ± 0.05	1.69 ± 0.09
Left kidney weight/100 g BW	0.32 ± 0.01	0.34 ± 0.01	0.31 ± 0.01	0.3 ± 0.01	0.31 ± 0.01
Liver weight (g)	16.1 ± 0.6	16.8 ± 0.5	17.7 ± 0.7	18.3 ± 0.8	16.2 ± 1.0
Liver weight/100 g BW	3.04 ± 0.06	3.25 ± 0.05	3.39 ± 0.17	3.53 ± 0.19	3.04 ± 0.2
Systolic blood pressure (mmHg)	147 ± 3	158 ± 2^∗^	160 ± 3^∗^	172 ± 1^∗#†^	152 ± 2^‡^

^∗^ *P* < 0.05 versus control; ^#^*P* < 0.05 versus DEX; ^†^*P* < 0.05 versus HF; ^‡^*P* < 0.05 versus DEX + HF.

**Table 3:** Plasma l-citrulline, l-arginine, and dimethylarginine levels.

	Control	DEX	HF	DEX + HF	DEX + HF + DMF
l-Citrulline	62.3 ± 4.6	65 ± 5.1	65.1 ± 5.9	70.6 ± 6.2	62.6 ± 3.3
l-Arginine	319.9 ± 35	315.5 ± 28.3	283.8 ± 24.7	289 ± 25.8	343.8 ± 30.1
ADMA	1.78 ± 0.4	2.32 ± 0.23	2.39 ± 0.2	2.72 ± 0.09^∗^	1.66 ± 0.28^#‡^
SDMA	1.42 ± 0.29	1.82 ± 0.17	1.88 ± 0.15	2.11 ± 0.07^∗^	1.33 ± 0.21^#‡^
l-Arginine-to-ADMA ratio	226 ± 50	153 ± 37	119 ± 7^∗^	107 ± 9^∗^	253 ± 63^‡^

^∗^ *P* < 0.05 versus control; ^#^*P* < 0.05 versus DEX; ^‡^*P* < 0.05 versus DEX + HF.
